# Analysis of survival rate and persistence predictors of baricitinib in real-world data from a large cohort of rheumatoid arthritis patients

**DOI:** 10.1016/j.crphar.2024.100178

**Published:** 2024-02-16

**Authors:** Simone Parisi, Becciolini Andrea, Ditto Maria Chiara, Alberto Lo Gullo, Larosa Maddalena, Scolieri Palma, Addimanda Olga, Reta Massimo, Marino Paroli, Caccavale Rosalba, Visalli Elisa, Foti Rosario, Amato Giorgio, De Lucia Francesco, Dal Bosco Ylenia, Foti Roberta, Farina Antonella, Girelli Francesco, Bernardi Simone, Camellino Dario, Bianchi Gerolamo, Colina Matteo, Andracco Romina, Mansueto Natalia, Ferrero Giulio, Del Medico Patrizia, Molica Colella Aldo, Franchina Veronica, Molica Colella Francesco, Lumetti Federica, Sandri Gilda, Salvarani Carlo, Priora Marta, Ianniello Aurora, Nucera Valeria, Santilli Daniele, Lucchini Gianluca, Giuditta Adorni, Di Donato Eleonora, Bravi Elena, Platè Ilaria, Arrigoni Eugenio, Bezzi Alessandra, Focherini Maria Cristina, Mascella Fabio, Bruzzese Vincenzo, Ravagnani Viviana, Fiorenza Alessia, Rovera Guido, Vitetta Rosetta, Marchetta Antonio, Volpe Alessandro, Ometto Francesca, Ariani Alarico, Fusaro Enrico

**Affiliations:** a Azienda Ospedaliero Universitaria Città della Salute e della Scienza di Torino, Rheumatology Unit, Turin, Italy; b Azienda Ospedaliero-Universitaria di Parma, Department of Medicine, Internal Medicine and Rheumatology Unit, Parma, Italy; c Azienda Ospedaliera di Rilievo Nazionale e di Alta Specializzazione Garibaldi Ospedale Garibaldi-Nesima, Rheumatology Unit, Catania, Italy; d Azienda Sanitaria Locale 3 Genovese, Division of Rheumatology - Medical Specialties Department, Genoa, Italy; e Ospedale Nuovo Regina Margherita, Internal Medicine and Rheumatology Unit, Rome, Italy; f IRCCS Azienda Ospedaliero-Universitaria di Bologna Policlinico S Orsola, Department of Internal Medicine-Rheumatology, Bologna, Italy; g University of Rome La Sapienza, Department of Clinical, Anesthesiological and Cardiovascular Sciences, Polo Pontino, Latina, Italy; h Azienda Ospedaliero Universitaria Policlinico Vittorio Emanuele Catania, Division of Rheumatology, A.O.U. “Policlinico San Marco”, Catania, Italy; i ASUR Area Vasta 4 Fermo, Ospedale A Murri, Internal Medicine Unit, Rheumatology outpatient clinic, Fermo, Italy; j Morgagni-Pierantoni Hospital, Rheumatology Unit, Forlì, Italy; k Azienda USL di Imola, Department of Internal Medicine and Oncology. Service of Rheumatology, Imola, Italy; l University of Bologna, Department of Biomedical and Neuromotor Sciences, Imola, Italy; m Hospital Santa Corona Pietra Ligure, Internal Medicine Unit, Rheumatology outpatient clinic, Unit of Diagnostic and Interventional Radiology, Pietra Ligure, Italy; n Civitanova Marche Hospital, Rheumatology outpatient clinic, Internal Medicine Unit, Civitanova Marche, Italy; o Azienda Ospedaliera Papardo Piemonte, Rheumatology Unit, Messina, Italy; p Azienda Ospedaliera Papardo Piemonte, Oncology Unit, Messina, Italy; q Università degli Studi di Milano-Bicocca, Internal Medicine Unit, Milan, Italy; r University Hospital Modena, Rheumatology Unit, Modena, Italy; s University of Modena and Reggio Emilia, Rheumatology Unit, Modena and Reggio Emilia, Italy; t ASL 15 Cuneo, Rheumatology Day Hospital and outpatient clinic, Mondovì, Italy; u ASL 13 Novara, Rheumatology Outpatient Unit, Novara, Italy; v Guglielmo da Saliceto Hospital, Department of Medicine, Internal Medicine and Rheumatology Unit, Piacenza, Italy; w ASL 13 Rimini, Internal Medicine and Rheumatology Unit, Rimini, Italy; x Santa Chiara Hospital of Trento, Rheumatology Unit, Trento, Italy; y PO S Andrea di Vercelli, Unit of Rheumatology, Vercelli, Italy; z IRCCS Ospedale Sacro Cuore Don Calabria, Rheumatology Unit, Negrar, Italy; aa Azienda ULSS 6 Euganea, Rheumatology Outpatient Clinic, Padova, Italy

**Keywords:** JAK inhibitors, tsDMARD, bDMARD, Rheumatoid arthritis, Survival rate, Baricitinib, Comorbidity, Prognostic factor, Real world, Line of treatment

## Abstract

**Objectives:**

The persistence in therapy of rheumatoid arthritis drugs and particularly bDMARD is a limiting factor for their long-term use. The randomized controlled trials (RCTs) may not reflect real-world contexts due to strict inclusion and exclusion criteria. Baricitinib, which targets both JAK1 and JAK2, has been used in Italy for several years. The aim of this multi-center study is to assess the real world persistence on therapy of baricitinib in RA patients and to identify predictive factors of baricitinib's survival rate.

**Methods:**

This is a retrospective, multicentric, Italian, longitudinal study. All patients were enrolled according to the following criteria: a) age ≥ 18 years old; b) diagnosed with RA according 2010 ACR/EULAR classification criteria; c) treated with baricitinib. In order to describe baricitinib clinical efficacy, the survival rate was evaluated by The Kaplan–Meier curve. Then, predictive factors of drug retention rate were assessed by performing the Cox analysis, identifying which risk factors influenced treatment persistence.

**Results:**

Overall, we included 478 patients treated with baricitinib. Among them, 380 (79.5%) were females. Baricitinib's survival rate was 94.6% at 6 months, 87.9% at 12 months, 81.7% at 24 months and 53.4% at 48 months. The Cox analysis regression showed that a higher bDMARDs/tsDMARD line of therapy seems to be a negative prognostic factor for the drug retention rate (HR 1.26 CI 95% 1.07–1.49, p = 0.006.

**Conclusion:**

Real-life study confirms baricitinib effectiveness up to 4 years, but previous treatment with bDMARDs was a negative prognostic factor for its survival rate.

## Highlights

• This is the first multi-center, real-life study in Italy, examining RA (Rheumatoid Arthritis) patients treated with baricitinib over a prolonged follow-up period.
• The study demonstrated a good retention rate of baricitinib over 4 years compared to other cohorts and real-world data of other JAK inhibitors.
• This study allows to analyze the predictive factors of persistence in baricitnib therapy, also analyzing a population of elderly patients.
• A higher number of previous bDMARD treatments is a negative predictive factor for barictinib's retention rate.

## Graphical abstract

**Figure:**
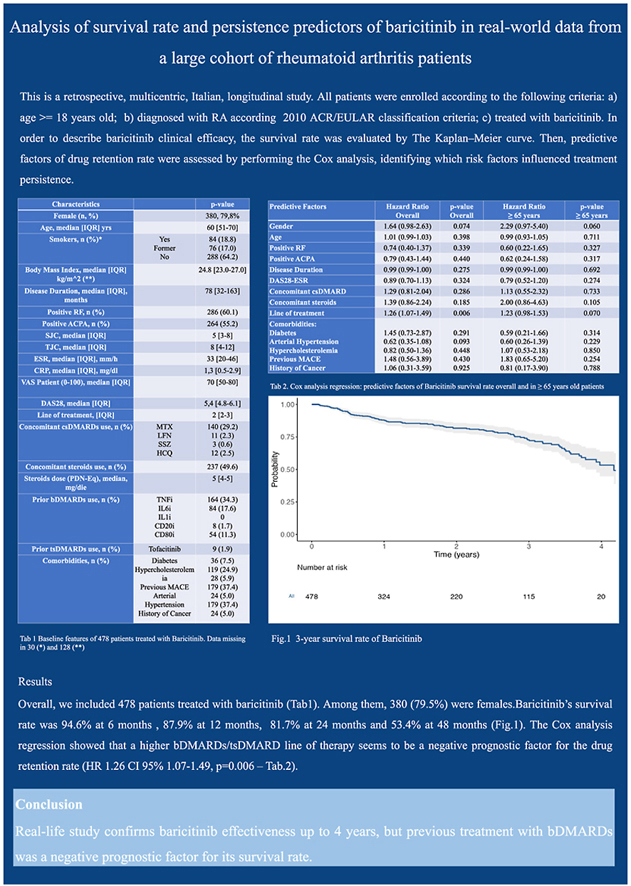

## 1 Introduction

In recent years, therapeutic strategies for Rheumatoid Arthritis (RA) have shifted towards a more personalized approach based on the treat-to-target principle, using modified antirheumatic drugs (DMARDs) to achieve remission or low disease activity (LDA) (Smolen et al., 2023; Singh et al., 2016; Lau et al., 2019).

Janus kinases (JAKs) play a crucial role in the signaling pathways of various cytokines involved in the development of RA. The JAK family consists of four cytoplasmic protein tyrosine kinases: JAK1, JAK2, JAK3, and Tyk2. Hence, due to their involvement in cytokine signaling, JAKs have emerged as a potential therapeutic target for RA (Choy et al., 2019; Silvagni et al., 2020).

Four JAK inhibitors (baricitinib, tofacitinib, upadacitinib, and filgotinib), have been approved for RA treatment and are considered a new class of targeted synthetic DMARDs (tsDMARDs). Current guidelines rank them on the same level as biological DMARDs (bDMARDs) after conventional synthetic DMARDs (csDMARDs) have failed (Smolen et al., 2023).

Baricitinib is an oral tsDMARD that targets JAK1 and JAK2, and it is involved in the regulation of various RA cytokine pathways (Choy et al., 2019). Since its approval in Europe in 2018, its efficacy and safety have been tested in several randomized clinical trials (RCTs) (Dougados et al., 2017; Genovese et al., 2016; Fleischmann et al., 2017) in RA patients who have failed csDMARDs or bDMARDs. In particular, baricitinib has shown good results, when compared to adalimumab and methotrexate (MTX) in csDMARD insufficient responders (IR) patients (Taylor et al., 2017).

While RCTs are tailored to specific population of patients who can be enrolled in such studies by applying very strict inclusion and exclusion criteria, thus they should maximize bias reduction and confounding factors. Nevertheless, it is commonly ascertained that patients enrolled in such trial may do not represent the real life context. Indeed the reduce bias and confounding factors through randomization and the use of strict inclusion and exclusion criteria, the patients included are not typically representative of a real-world context (Kim et al., 2018). In addition, observations from routine clinical practice, can provide reliable and reproducible information (Egger et al., 2016; Roche et al., 2013).

In contrast to other JAKis (tofacitinib), to date only scanty data are available on baricitinib (Baldi et al., 2023; Guidelli et al., 2021; Spinelli et al., 2021; Tesei et al., 2021; Perrone et al., 2020).

Therefore, the main aim of this multi-center study is to assess the survival rate of baricitinib in a real life cohort of RA patients. The secondary aim consist in identifying predictive factors of baricitinib's survival rate.

## 2 Methods

This is a retrospective, multicentric, Italian, longitudinal study carried out in 26 rheumatology and internal medicine units. All patients were enrolled according to the following criteria: a) age ≥ 18 years old; b) diagnosed with RA according 2010 ACR/EULAR classification criteria (Aletaha et al., 2010); c) treated with baricitinib.

For each patient, we collected the following characteristics: gender (female/male), age (years), disease duration (months), rheumatoid factor (RF), positive anti-citrullinated proteins antibodies (ACPA), concomitant treatment including (cs)DMARDs, previous treatments with biological (bDMARDs) or tsDMARDs, disease activity assessed by DAS28-ESR.

The protocol was approved by Ethics Committee “Comitato Etico Interaziendale AOU Città della Salute e della Scienza di Torino – AO Ordine Mauriziano di Torino – ASL Città di Torino” with number 524/2021 on Dec 20, 2021.

## 3 Statistical analysis

All numeric variables were reported by median value and interquartile range (IQR) if continuous or as percentage if categorical.

In order to describe baricitinib clinical efficacy, the survival rate was evaluated by The Kaplan–Meier curve. Then, predictive factors of drug retention rate were assessed by performing the Cox analysis, identifying which risk factors influenced treatment persistence (age, gender, disease duration, relevant comorbidity, baseline DAS28-ESR, concomitant steroid or csDMARDs treatment, line of bDMARDs/tsDMARDs treatment).

A p-value<0.05 was considered statistically significant. All statistical analyses were performed with Jamovi software (https://www.jamovi.org, ver .2.3.22).

## 4 Results

Overall, we included 478 patients treated with baricitinib. Among them, 380 (79.5%) were females. 286 (60.1%) patients presented a positive RF and 264 (55.2%) positive ACPA. All the baseline features of this cohort are summarized in Table 1. In 105 (22.0%) patients, baricitinib was prescribed as first line treatment after csDMARDS, the remaining 363 patients (75.1%) had failed at least one bDMARD and 9 (1.9%) also failed a tsDMARD. In 34.7% of cases baricitinib was used in monotherapy, and when used in combo therapy, the most frequently associated csDMARD was methotrexate (29.2%). The median survival rate period was 674 days (298–1087).

**Table 1:** Baseline features of 478 patients treated with Baricitinib. Data missing in 30 (*) and 128 (**) patients.

Characteristics		p-value
Female (n, %)		380, 79,8%
Age, median [IQR] yrs		60 [51–70]
Smokers, n (%)*	Yes	84 (18.8)
	Former	76 (17.0)
	No	288 (64.2)
Body Mass Index, median [IQR] kg/m^2 (**)		24.8 [23.0–27.0]
Disease Duration, median [IQR], months		78 [32–163]
Positive RF, n (%)		286 (60.1)
Positive ACPA, n (%)		264 (55.2)
SJC, median [IQR]		5 [3–8]
TJC, median [IQR]		8 [4–12]
ESR, median [IQR], mm/h		33 [20–46]
CRP, median [IQR], mg/dl		1,3 [0.5–2.9]
VAS Patient (0–100), median [IQR]		70 [50–80]
DAS28, median [IQR]		5,4 [4.8–6.1]
Line of treatment, [IQR]		2 [2–3]
Concomitant csDMARDs use, n (%)	MTX	140 (29.2)
	LFN	11 (2.3)
	SSZ	3 (0.6)
	HCQ	12 (2.5)
Concomitant steroids use, n (%)		237 (49.6)
Steroids dose (PDN-Eq), median, mg/die		5 [4–5]
Prior bDMARDs use, n (%)	TNFi	164 (34.3)
	IL6i	84 (17.6)
	IL1i	0
	CD20i	8 (1.7)
	CD80i	54 (11.3)
Prior tsDMARDs use, n (%)	Tofacitinib	9 (1.9)
Comorbidities, n (%)	Diabetes	36 (7.5)
	Hypercholesterolemia	119 (24.9)
	Previous MACE	28 (5.9)
	Arterial Hypertension	179 (37.4)
	History of Cancer	24 (5.0)

RF, Rheumatoid Factor; ACPA, Anti-Citrullinated Proteins Antibodies; SJC, swollen joints count; TJC, tender joints count; ESR, erythrocyte sedimentation rate; CRP, C-reactive protein, VAS, Visual Analogic Scale; DAS28, Disease Activity Score 28; csDMARD, conventional synthetic modified antirheumatic drugs; PDN-Eq, prednisone equivalent, bDMARD, biological modified antirheumatic drugs; tsDMARD, terget synthetic modified antirheumatic drugs. TNFi, TNF inhibitors; IL-6i, IL-6 inhibitors; IL-1i, IL-1 inhibitors, CD20i, CD20 inhibitors, CD80i, CD80inhibitors; MACE, Major Adverse Cardiovascular Events.

**Fig. 1:**
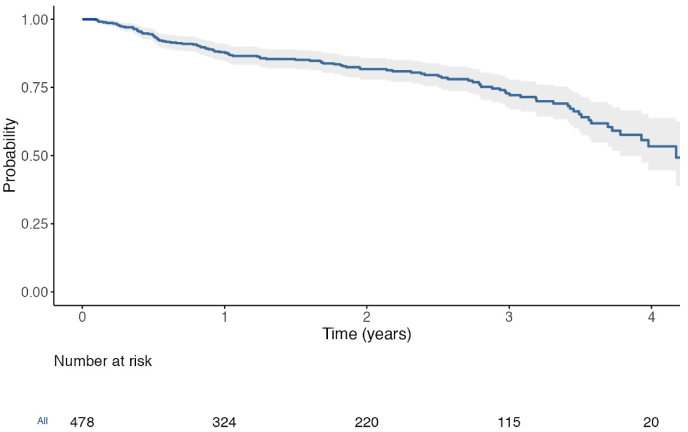
4-year survival rate of Baricitinib.

Baricitinib's survival rate was 94.6% at 6 months, 87.9% at 12 months, 81.7% at 24 months and 53.4% at 48 months (Table 2, Fig. 1).

**Table 2:** Retention rate of Baricitinib and Tofacitinib in real world studies.

	Study	6 months	12 months	18 months	24 months	36 months	48 months
**Baricitinib % of retention rate**	Parisi et al.	94.6	87.89		81.7	72.5	53.4
	Hernández-Cruz et al. (Hernández-Cruz et al., 2022)	79.7	64.8	59.1			
	Iwamoto et al. (Iwamoto et al., 2021)	81.5	–	–	–	–	–
	Baldi et al. (Baldi et al., 2023)		75.1		69.3		
**Tofacitinib % of retention rate**	Iwamoto et al. (Iwamoto et al., 2021)	76.4	–	–	–	–	–
	Tamura N (Tamura et al., 2018)	77.3	–	–	–	–	–
	Pope J (Pope et al., 2020)	–	62.7	–	49.6	–	–
	Mori et al. (Mori and Ueki, 2019)	–	68	–	–	–	–
	Zengin et al. (Zengin et al., 2018)	–	75	–	–	48	–
	Bilgin et al. (Bilgin et al., 2020)	–	64	–	–	–	–
	Movahedi et al. (Movahedi et al., 2020)	–	–	64	–	–	–

Overall, discontinuation of baricitinib was due to: lack of efficacy (n = 35), loss of efficacy (n = 20), infections (n = 8) and venous thromboembolism VTE (n = 5).

The Cox analysis regression showed that a higher bDMARDs/tsDMARD line of therapy seems to be a negative prognostic factor for the drug retention rate (HR 1.26 CI 95% 1.07–1.49, p = 0.006. All the other variables assessed did not result significantly associated to baricitinib survival rate (Table 3). In addition, the same analysis was applied to patients ≥65 years of age (181/476, 38%). In this instance, none of the variables analyzed was statistically significant on the impact of the retention rate (Table 3).

**Table 3:** Cox analysis regression: predictive factors of Baricitinib survival rate overall and in ≥65 years old patients.

Predictive Factors	Hazard Ratio Overall	p-value Overall	Hazard Ratio ≥65 years	p-value ≥65 years
Gender	1.64 (0.98–2.63)	0.074	2.29 (0.97–5.40)	0.060
Age	1.01 (0.99–1.03)	0.398	0.99 (0.93–1.05)	0.711
Positive RF	0.74 (0.40–1.37)	0.339	0.60 (0.22–1.65)	0.327
Positive ACPA	0.79 (0.43–1.44)	0.440	0.62 (0.24–1.58)	0.317
Disease Duration	0.99 (0.99–1.00)	0.275	0.99 (0.99–1.00)	0.692
DAS28-ESR	0.89 (0.70–1.13)	0.324	0.79 (0.52–1.20)	0.274
Concomitant csDMARD	1.29 (0.81–2.04)	0.286	1.13 (0.55–2.32)	0.733
Concomitant steroids	1.39 (0.86–2.24)	0.185	2.00 (0.86–4.63)	0.105
Line of treatment	**1.26 (1.07–1.49)**	**0.006**	1.23 (0.98–1.53)	0.070
Comorbidities:				
Diabetes	1.45 (0.73–2.87)	0.291	0.59 (0.21–1.66)	0.314
Arterial Hypertension	0.62 (0.35–1.08)	0.093	0.60 (0.26–1.39)	0.229
Hypercholesterolemia	0.82 (0.50–1.36)	0.448	1.07 (0.53–2.18)	0.850
Previous MACE	1.48 (0.56–3.89)	0.430	1.83 (0.65–5.20)	0.254
History of Cancer	1.06 (0.31–3.59)	0.925	0.81 (0.17–3.90)	0.788

RF, Rheumatoid Factor; ACPA, Anti-Citrullinated Proteins Antibodies; DAS28, Disease Activity Score 28; csDMARD, conventional synthetic modified antirheumatic drugs; MACE, Major Adverse Cardiovascular Events.

## 5 Discussion

This is the first multi-center, Italian, real life study carried out in a cohort of RA patients treated with baricitinib for a long follow up period.

Our data showed a good retention rate of baricitinib over 4 years of follow up compared to other cohort and other JAKi's real world data ((Hernández-Cruz et al., 2022; Iwamoto et al., 2021; Tamura et al., 2018; Pope et al., 2020; Mori and Ueki, 2019; Zengin et al., 2018; Bilgin et al., 2020; Movahedi et al., 2020; Baldi et al., 2023), Table 3).

In line with previous clinical trials and observational studies we observed a baseline high disease activity (DAS-ESR 5.4) (Dougados et al., 2017; Genovese et al., 2016; Taylor et al., 2017; Hernández-Cruz et al., 2022) and a similar median age at baseline (60 years old [51–60]). However, in contrast to previous studies, (Guidelli et al., 2021; Hernández-Cruz et al., 2022), our cohort had a lower disease duration (78 months [32–163]) and a lower seropositivity for RF and ACPA (RF positivity 60.1% and ACPA positivity 55.2% respectively).

In a recent retrospective study Baldi et al. (2023) assessed the retention rate of baricitinib in patients with rheumatoid arthritis. The results showed a good treatment persistence after 12 and 24 months of observation (75.1% and 69.3%, respectively). Combination with methotrexate did not influence persistence, but the use of steroids reduced treatment retention. Baricitinib therapy as the first-line treatment had a better retention rate compared to subsequent treatments. The use of steroids, their dosage, and previous treatments with bDMARDs increased the risk of treatment discontinuation. No significant adverse events were reported.

Accordingly with the aforementioned Italian study, regarding the Cox analysis, a worst retention rate was predicted by the line of treatment. As such, it seems that patients who have already experienced multiple lines of treatment present a more refractory disease; thus, multiple previous failure treatments could negatively impact on baricitinib efficacy (Nagy et al., 2021). Moreover, these patients may have a higher likelihood of developing side effects or drug interactions with other concomitant treatments (i. e., corticosteroids), which could consequently affect their persistence on baricitinib.

However, concomitan corticosteroid treatment does not result to impact on baricitinib'survival rate, suggesting baricitnib's efficacy regardless of the concomitant therapy. (Roodenrijs et al., 2021; Strehl et al., 2016).

In the Orbit Study (Hernández-Cruz et al., 2022) the better persistence was related to the use of baricitinib in combo-therapy, as already described for several bDMARDs (Gabay et al., 2015; Soliman et al., 2011; Heiberg et al., 2008; Zink et al., 2005; Kristensen et al., 2006). In our cohort, we did not find any association between combination treatment or monotherapy and retention rate, confirming that baricitinib is also effective in monotherapy (Ho Lee and Gyu Song, 2020; Fleischmann et al., 2020; Taylor et al., 2022).

Positive ACPA is a negative prognostic factor for RA, being associated with erosion and high irreversible damage. In a previous Italian study, carried out positive RF and ACPAwere associated with longer drug survival period (Guidelli et al., 2021). In our cohort we did not confirm this result. Indeed, Baricitinib acts differently than drugs that attempt to block the production of ACPA and directly targets the immune system and joint inflammation, regardless of the presence of these autoantibodies. What we have seen is more in agreement with RCT or pooled post hoc analyses (Wells et al., 2018).

Unlike what emerged in the Orbit study (Hernández-Cruz et al., 2022), where better persistence was related to lower Charlson, Comorbidity Index scores, our data did not show a significant correlation with any comorbidity; in addition, no impact on persistence was detected for variables such as age and gender.

A very interesting finding that emerged from our analysis is that the retention rate is not correlated with disease duration or baseline DAS28ESR This is very important because it suggests that the efficacy of baricitinib seems to be independent of the severity of the disease. This is very useful in clinical practice, especially in the treatment of patients who meet the criteria for D2T (Nagy et al., 2021).

The safety is a big concern about tsDMARDs and bDMARDs. Recently the European Medicines Agency's human medicines committee has endorsed measures recommended by the Pharmacovigilance Risk Assessment Committee to minimize the risk of serious side effects associated with JAK inhibitors, used to treat chronic inflammatory disorders. The measures include using these medicines with caution and reducing doses in patients with risk factors for blood clots, cancer, and major cardiovascular problems. The recommendations come after a review of available data, including the final results of a clinical trial and advice from an expert group of healthcare professionals and patient representatives (Wells et al., 2018). However, a thorough analysis of all RCT patients who were given baricitinib suggests that it has an acceptable safety profile when compared to bDMARDs (European Medicine Agency, 2023). A potential higher risk of thrombotic events has been reported for JAK inhibitors, and a post-marketing analysis of baricitinib trials estimated this risk to be small (about 5 events per 1000 patient years) and similar to the risk associated with rheumatoid arthritis itself (about 3–7 events per 1000 patient years) (Smolen et al., 2019; Scott et al., 2018). In our cohort we observed 5 thrombotic events (Table 4).

**Table 4:** Patients with venous thromboembolism during treatment with Baricitinib.

Pts	Age (y)	Gender	BMI	Smokers	Disease Duration (y)	Comorbidities	DAS28	Concomitant csDMARDs	Concomitant steroids
1	68	F	25,2	Former	15,8	D, AH, MACE	4,24	LFN	N
2	69	F	26,0	Former	8.3	AH, Hy	5,27	–	N
3	74	F	26	Former	11.6	AH	6,45	–	Y
4	61	F	27	Former	11.9	AH	5,67	–	Y
5	61	M	28,8	Former	5.3	D, AH	4,96	MTX	N

F, Female; M, Male; D, Diabetes; AH, Arterial Hypertension; Hy, Hypercholesterolemia, MACE, Previous Major Adverse Cardiovascular Events; csDMARD, conventional synthetic Disease Modifying Drug; LFN, leflunomide, MTX, Methotrexate, N, no; Y, yes.

Finally, on the basis of what was published by the EMA on patients at risk treated with JAK-i, we carried out the analysis of the predictive factors of response also on the population ≥65 years old patients. There were no substantial differences with respect to the predictive factors already emerged and in this group of patients the line of treatment does not seem to have an impact either. It is known in the literature how the phenomenon of immunosenescence can correlate with inflammation and how advanced age can be a greater risk of strengthening inflammation levels (Chalan et al., 2015; Taylor et al., 2019; Covre et al., 2020). Despite all the limitations of the study, it is possible to explain this data as an effect of treatment with the JAK-i not only on the reduction of inflammatory phenomena directly mediated by rheumatoid arthritis, but also by immunosenescence (Xu et al., 2015).

## 6 Study limitations

However, this descriptive study does have some limitations. Firstly, its retrospective design and consequently, missing data. In addition, the generalizability of the results is limited by the geographical variation in routine clinical practice, and the lack of a comparator group makes it difficult to determine how the various assessed variables compare to other treatments.

Nevertheless, it is noteworthy that our cohort include a large sample of RA patients, and a quite long follow up period of 4 years, which is not common, compared to previous observational studies (Guidelli et al., 2021; Spinelli et al., 2021; Tesei et al., 2021; Perrone et al., 2020; Hernández-Cruz et al., 2022; Baldi et al., 2023).

## 7 Conclusion

This study provides evidence for the persistence of baricitinib up to 4 years in a real-life setting that appears consistent with reports from the pivotal studies. Furthermore, from this preliminary experience, predictors of retention rate to baricitinib therapy have been identified and there were also confirmed in older patients Seropositivity and combo therapy seems to not correlate with a better retention rate, while line of treatment is a negative prognostic factor.

In the absence of studies with a larger sample size and longer follow-up period, these real-world data provide the best available evidence to aid rheumatologists in the therapeutic management of these patients.

## 8 Key messages

1. This is real-world study of a large cohort of RA patients treated with baricitinib with a long observation period.
2. This study allows to analyze the predictive factors of persistence in baricitnib therapy, also analyzing a population of elderly patients.
3. A higher number of previous bDMARD treatments is a negative predictive factor for barictinib's retention rate.

## Funding

This study was conducted without external funding or financial support.

## Declaration of competing interest

The authors declare that they have no known competing financial interests or personal relationships that could have appeared to influence the work reported in this paper.

## Data Availability

Data will be made available on request.
